# Village health workers as health diplomats: negotiating health and study participation in a malaria elimination trial in The Gambia

**DOI:** 10.1186/s12913-021-07431-y

**Published:** 2022-01-11

**Authors:** Yoriko Masunaga, Joan Muela Ribera, Fatou Jaiteh, Daniel H. de Vries, Koen Peeters Grietens

**Affiliations:** 1 Unit of Socio-Ecological Health Research, Department of Public Health, Institute of Tropical Medicine, Antwerp, Belgium; 2 Faculty of Social and Behavioural Science, Department of Sociology and Anthropology, University of Amsterdam, Amsterdam, The Netherlands; 3 PASS Suisse, Neuchâtel, Switzerland; 4 Medical Anthropology Research Centre at Departament d’Antropologia, Filosofia i Treball Social, Universitat Rovira i Virgili, Tarragona, Spain; 5 Medical Research Council Unit the Gambia at the London School of Hygiene and Tropical Medicine, Fajara, The Gambia; 6 School of Tropical Medicine and Global Health, Nagasaki University, Nagasaki, Japan

**Keywords:** Village health workers, Community participation, Health diplomat, Symbolic capital, Capability approach, Malaria elimination trial, The Gambia

## Abstract

**Background:**

Although many success stories exist of Village Health Workers (VHWs) improving primary health care, critiques remain about the medicalisation of their roles in disease-specific interventions. VHWs are placed at the bottom of the health system hierarchy as cheap and low-skilled volunteers, irrespective of their highly valued social and political status within communities. In this paper, we shed light on the political role VHWs play and investigate how this shapes their social and medical roles, including their influence on community participation.

**Method:**

The study was carried out within the context of a malaria elimination trial implemented in rural villages in the North Bank of The Gambia between 2016 and 2018. The trial aimed to reduce malaria prevalence by treating malaria index cases and their potentially asymptomatic compound members, in which VHWs took an active role advocating their community and the intervention, mobilising the population, and distributing antimalarial drugs. Mixed-methods research was used to collect and analyse data through qualitative interviews, group discussions, observations, and quantitative surveys.

**Results and discussion:**

We explored the emic logic of participation in a malaria elimination trial and found that VHWs played a pivotal role in representing their community and negotiating with the Medical Research Council to bring benefits (e.g. biomedical care service) to the community. We highlight this representative role of VHWs as *‘health diplomats’*, valued and appreciated by community members, and potentially increasing community participation in the trial. We argue that VHWs aspire to be politically present and be part of the key decision-makers in the community through their health diplomat role.

**Conclusion:**

It is thus likely that in the context of rural Gambia, supporting VHWs beyond medical roles, in their social and political roles, would contribute to the improved performance of VHWs and to enhanced community participation in activities the community perceive as beneficial.

## Introduction

The declaration of Alma-Ata highlighted the importance of primary health care and the community’s “full participation” to bring health care close to the ground [1]. In response, many governments in low- and middle-income countries adopted lay health worker programmes [2] to the point that lay health workers have become synonymous with primary health care [3]. Over time, the term lay health worker has come to represent various names such as community health worker, community health agent, village health worker, health promoter, and many more [4]. Broadly, a lay health worker is “a member of the community who has received some training to promote health or to carry out some health-care services, but is not a health-care professional” p.919 [5]. In general, they are expected to take medical and social roles: the medical role handles biomedical tasks such as delivering diagnostic and treatment services; and the social role mainly involves mobilising and communicating with the population [6]. Globally there are many success stories of lay health workers contributing to improved primary health care in a cost-effective way [6–8]. For example in The Gambia, a number of studies have shown that effective performance of lay health workers reduced child mortality and also effectively provided malaria preventive treatment to infants [9–12].

However, there have been longstanding critiques that lay health workers are, by design, conceived as ‘cost-effective’ and lowly skilled interfaces between under-resourced health systems and the communities [13–15], and posited at the bottom of the health system pyramid and medical intervention hierarchies [15]. In fact they can be used as cheap front-line medical workers or decorative forms of ‘community participation’ for vertical interventions [15–17]. It is partly due to the dependent structure where capacity and success of lay health worker programmes are mostly attributed to international donor programmes [2, 6], without serious integration of local governments which are unlikely to have the capacity to take over and sustain such programmes [18]. Despite the global push to promote primary health care and its sustainability, the dependency remains, and lay health workers remain ‘cheap labours’ without sufficient funds made available for them. This inadequate support for lay health workers contributes to higher attrition, which has been identified as a challenge of primary health care [5].

To understand and improve lay health worker programmes, numerous studies have investigated their social and medical roles and their unique position as the bridge between the health system and the communities. Some studies highlight positive health outcomes linked to social networks, norms, and trust that surround lay health workers [19–23]. Other studies underline incentive structures that drive lay health workers to perform their duties, such as: expectations to gain financial and material incentives, technical supervisions, job opportunities, social recognition, and trust from communities and health systems [24–29]. Additionally, there is a growing trend to promote entrepreneurship amongst lay health workers [30].

However, lay health worker relationships to their communities remain somewhat unclear [31], despite their “community embeddedness” has been emphasised as a requirement to serve communities and to assure successful community participation in health interventions [16]. Moreover, there is a lack of attention concerning lay health workers’ political role and status that may shape their social and medical roles. Only in the American context lay health workers have been described as social justice and policy advocates in addition to their role as health promoters and social agents [32–35]. In this instance, lay health workers who previously had leadership or advocacy training were likely to advocate at the political and civic level [32, 33] and inform policies based on communities’ realities [34] as a ‘broker’ between the poor (in community) and the state [14]. Finally, it has been long warned that by failing to truly understand lay health workers’ roles, resources, expectations and relationships to communities, primary health care could become a system of mass production of lay health workers without consideration of their usefulness or impacts [36].

In this paper, we explore the role of lay health workers, namely Village Health Workers (VHWs) in The Gambia and assess their political role as well as their social and medical roles. We aim to answer *how VHWs political role and status shape their social and medical roles, and consequently influence the community’s participation in health interventions such as malaria elimination programmes.* To understand to what extent, how, and why VHWs’ status is valued in the community, we draw upon the analysis of their symbolic capital – a form of capital that is produced and reinforced within a group of people believing its quality and value, such as prestige and reputation [37, 38] – that may affect people’s perception or relationship towards VHWs. We further explore, through Sen’s capability approach [39], VHWs’ capability to influence community participation and to pursue community’s healthy living.

## Methods

### VHW in a malaria elimination trial

In The Gambia, VHWs serve a village of a minimum of 400 people to provide maternal services, child health services, health education, promotion, and treatment of common illnesses, including malaria [40]. This paper presents the results of social science study which closely worked with 10 VHWs involved in the cluster-randomised trial, Reactive Household-based Self-administered Treatment against residual malaria transmission (RHOST). All of them were male farmers and/or herders, with similar socio-economic status as other community members. Most of them had not completed formal education, while a few had never been to a formal school (excepting Koranic school). Only the 3 VHWs who had (almost) completed primary education were able to communicate in English.

RHOST trial (registered with ClinicalTrials.gov, NCT02878200) was conducted by the Medical Research Council unit in The Gambia (MRCG) between 2016 and 2018, in 34 villages (randomly split for intervention and control) in the North Bank region of The Gambia. The trial aimed to reduce malaria prevalence in the study villages by treating malaria cases and their potentially asymptomatic compound members [41]. The trial provided additional training and supply of malaria diagnostic and treatment tools to VHWs beyond the national health system scheme [42]. The VHWs role in the trial intervention arm was mainly to diagnose and treat malaria index cases, prescribe and distribute antimalarial drugs to compound members of the index case, and communicate with community members and with the trial team. This role of VHWs was shaped by community members participating in the Community Lab of Ideas for Health (CLIH) – a specific participatory approach developed and conducted within the trial [43]. In CLIH, the trial and communities co-developed implementation strategies in which VHWs became the important trial implementors because of their highly regarded social and political status and of trust by community members (see results). The trial provided each VHW with a monthly monetary incentive (1500Dalasi = ±25EUR, equivalent to a 50 kg bag of rice) during the implementation period (i.e. malaria season around June to December) for the VHWs to take up this additional role on top of their routine roles for primary health care.

### Study population

The population comprised mainly Mandinka, Fula, and Wolof ethnicities while also including Bambara, Turka, and Tilibonka minorities. There were almost no mono-ethnic group village but a combination of different ethnic groups residing together harmoniously. Inter-ethnic marriage was common [44]. These societies were polyglot while each has its own language. The population was Muslim and mostly farmers (for both self-consumption and cash-crop) and/or herders. Many villages were located far from the main road and health facilities, making access to health services difficult. In the villages, the social organisation was based on patrilineal kinship [45]. The head of the village was the *Alkalo*, a role traditionally inherited patriarchally from the village founder who was surrounded and advised by the council of elders consisting of an Imam, deputy-Imam, Marabout (religious teacher and healer), and elderly compound heads. Village administration was managed by the Village Development Committee (VDC) who were responsible for development activities in the community. The VDC comprised of VHW, sub-committees such as woman’s groups, youth groups, and a representative from each ethnic group. A compound head was responsible for his compound members consisting of his family, extended family members, and sometimes guests and/or seasonal workers, and was regarded as the role-model in the family [45–47].

### Study design

A social science study was conducted within the trial, using a sequential exploratory mixed-method study design (QUAL-quan). Social science study aimed to provide contextual information to the trial and aid in co-creating trial implementation strategies with communities [43]. The field research team consisted of researchers and local fieldworkers with diverse backgrounds and experiences.

### Qualitative strands

#### Data collection

The field research team conducted a total of 161 in-depth interviews, 93 focus group discussions (including exploratory participatory workshops, key-informants’ meetings, monitoring meetings), 160 monitoring calls, and observations with informal chats by visiting and staying in intervention villages (*n* = 17) between March 2016 and December 2017. We developed a topic guide prior to data collection and adjusted it accordingly to emerging findings and hypotheses. Interviews took place at respondents’ convenience mostly in informal settings such as one’s household. Discussions were held by appointment – we consulted the *Alkalo* and VHW for the best available date (e.g. Friday after prayer time) and invited pre-identified key-informants to join the discussions. All conversations were translated from local languages (Mandinka, Fula, and Wolof) to English and vice versa by the local fieldworkers. Semi-structured monitoring calls were made to VHWs by fieldworkers twice a month during the implementation. Additionally, as part of our observations, we carefully observed both everyday village life and VHW life. The first included hierarchies among household/compound members, neighbours, visitors, youth and the elderly, available malaria protective measures, day-to-day socio-economic activities, and mobility; while the latter included observations on VHWs’ relation to other community members, their performances during VHWs’ training (*n* = 2), performance drills (*n* = 10), and actual patient visits.

#### Sampling

We purposefully selected our respondents and carried out snowball sampling. Our respondents were mainly: VHWs, traditional birth attendants, *Alkalos*, Imams, traditional healers, compound heads, members of VDC including women and youth groups, teachers, farmers, and caretakers (often women) of malaria patients. Participants for the discussions were identified through the ongoing ethnographic study, which was then followed by a stakeholder analysis.

#### Analysis

We analysed data concurrently during data collection, through ongoing field analysis with the research team discussing and validating findings to minimise possible bias, as well as iteratively making and testing various hypotheses. Field notes were transferred into digital form immediately after data collection every day. Most audio-recorded conversations were transcribed verbatim in English by the fieldworkers. We used NVivo (ver.11) qualitative data analysis software to code transcribed interviews and notes.

### Quantitative strands

#### Data collection

Two rounds of survey administration took place in June 2016 (baseline) and November–December 2017 (endline) in both intervention (*n* = 17) and control villages (n = 17) by the trained MRCG fieldworkers. The surveys were developed by the researchers based on the qualitative findings from the initial ethnographic study (March–May 2016). The surveys were paper-based containing both standardised closed- and open-ended questions. The surveys were first piloted to ensure clarity of questions and to avoid translation errors in local languages.

#### Sampling

Sample size was calculated by the trial epidemiologist ensuring that the number of households randomly selected was in proportion to the size of the village. A total of 324 baseline and 273 endline surveys were administered to adults above 16 years old (two samples of the same villages at different occasions and not among the same individuals). When any of the initially selected persons were unavailable, the next adult who shares similar characteristics (e.g. same-sex, closer in age) in the household was approached to complete the survey. When respondents were irreplaceable (no one to be found at a household), the non-response sheet was filled in. For this paper, we extracted the relevant questions related to VHWs (e.g. health-seeking behaviour, people’s perception towards VHWs) from the survey for intervention villages (baseline: *n* = 126, endline: *n* = 104) to focus on the baseline and endline differences in interventions villages. The size of the effective sample used in this study is thus 230 persons subjected to the trial intervention.

#### Analysis

Data were double entered by the trial data entry clerks. For this paper, a statistician calculated the frequencies, measures, and how likely these are compared with the expected distribution under randomness (the null hypothesis H_0_), with the Statistical Analysis System. The Chi-square test for two independent samples has been used, together with the Cramer’s V measure to have an idea of the strength of the relation between responses and baseline/endline variable for respondents in intervention villages [48]. Given the small number of cases (*n* = 230), we take 0.10 as the critical border (prob. < 0.10). For the analysis, we considered “NA (not applicable)” answer as a missing value and kept “DK (don’t know)” as a relevant response for analysis of people’s perception.

### Ethical consideration

All participants were provided an explanation, in their local language, of the study details and what it entailed prior to interviews, surveys, and discussions, as well as confidentiality and their rights to withdraw or not participate. Informed consent was obtained from all participants verbally, which was approved by The Gambia Government/MRC Joint Ethics Committee and the Institutional Review Board of the Institute of Tropical Medicine, Antwerp. Verbal consent was preferred due to the high rate of illiteracy among the study populations and to avoid sowing mistrust in communities by obliging signatures. We audio-recorded interviews and discussions with participant consent. All methods were carried out in accordance with relevant guidelines and regulations, including the Code of Ethics of the American Anthropological Association.

## Results and discussion

### Breaking down ‘participation’

Community participation is considered important for improved primary health care and VHWs are expected to mobilise local populations to participate in health programmes, and thus, we first explored the motivations of people to participate in the RHOST trial. Understanding communities’ interest (or disinterest) in participation helped us see for what VHWs represent and negotiate. Local terms mentioned in this section were explored in *Wolof*, the language the first-author and most of community members (regardless of their ethnicity) understand and speak. This enabled direct communication to better understand people’s reasonings of participation.

#### Individual and collective participation logic

[Participation] will not be forced because of the social cohesion (*juboo*) within us. So, when I make my call, people will be curious to know why I call them. That will make them answer to my call, but we don’t force anyone to come. – *Alkalo*, village-17

**Fig. 1:**
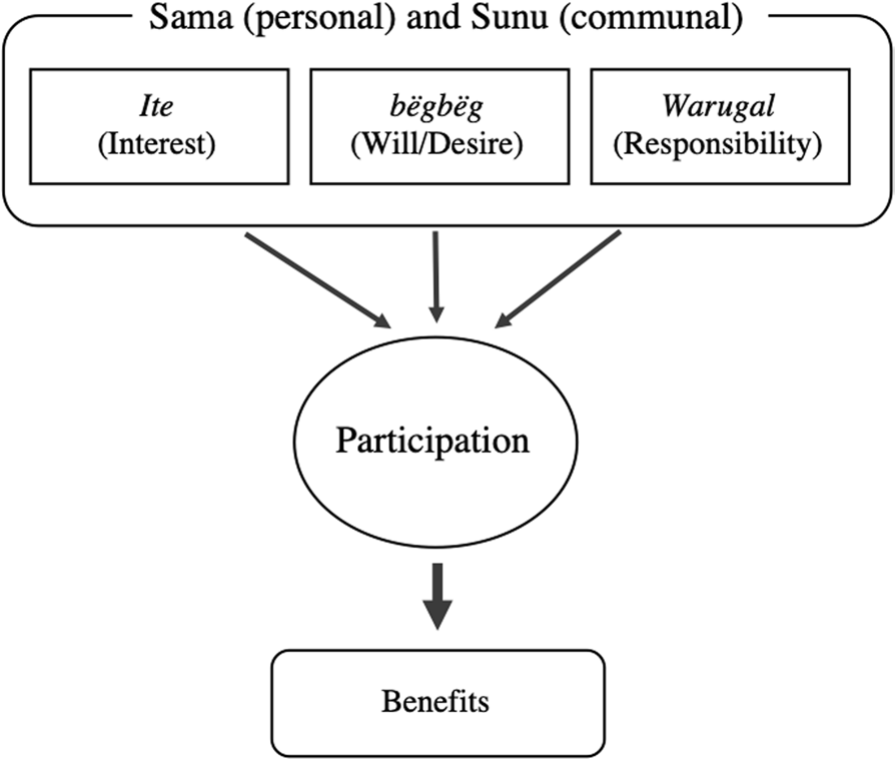
Emic logic of participation

In a society where social cohesion is an essential value, like in The Gambia, access and distribution of public goods are communal and shared with community members, and therefore, individual choice and action are expected to lead to collective benefits in addition to personal ones. According to our respondents, participation in a health intervention like RHOST happens when there is interest (*ite*), will/desire (*bëgbëg*), and/or obligation/responsibility (*warugal*). These elements of participation have personal ‘my’ (*sama*) and communal ‘our’ (*sunu*) aspects. The value of *sunu,* such as social cohesion (*juboo* – can also be translated as harmony), is considered necessary leverage for community participation, and is associated with increased participation in healthier acts [20, 49]. For example, taking medication to treat malaria is an individual (*sama*) action; however, it can be dependent on social (*sunu*) associations such as public perceptions, norms, responsibility and shared community value [50–52]. Following the local understanding, the logic of taking medication to treat malaria would be: my interest or willingness (*sama ite* or *bëgbëg*) to take antimalarial drugs contributes to the interruption of malaria transmission, which is in the community’s interest (*sunu ite*), and therefore, it is my/our responsibility (*sama/sunu warugal*) to participate in this activity and take the medicine that benefits the community’s health (Fig. 1).

Social norms can both directly and indirectly influence people’s participation. Individuals who refused to take medicine are considered selfish (*añan*), and what is considered ‘selfish (*añan*)’ or ‘desirable behaviour’ is internalised and normalised by disciplinary power. Actions are incited, induced, and seduced [53, 54], along with the direct and/or indirect sanctions [55, 56]. In our study, social pressure associated with social sanctions was observed, while an *Alkalo* said: *“participation will not be forced”*. For example, some people said they took antimalarial drugs otherwise they looked ‘bad’ [52].

In the rural Gambia, ‘good’ Gambians are those who share their resources (e.g. time, labour, or wealth) with communities, in alignment with Islam teaching of “giving (returning)” to the poorer [57]. Especially people with responsible roles (i.e. *Alkalo*, VHW, compound heads) have higher public expectations to act responsibly upon *sunu* to share benefits with the community, and in return, they receive symbolic capital, such as prestige and respect, from their community. Conversely, younger people acting according to *sama* is tolerated because of less expected responsibility. Case in point, our data on adherence to antimalarial drugs showed that young people adhered less [52]. As one’s role and social position change over time, individual capacity to influence people’s willingness and ability to participate shifts [58], with increasing emphasis on *sunu*.

#### Perceived benefits of participating in the MRCG trial

You (MRCG) are here to assist us. If the project stays here, it will benefit the whole village. – VHW, village-1

Staying healthy is considered everyone’s benefit in a place where sickness can lead to economic burden not only to oneself but to the whole compound [59–61]. When someone is sick, compound members sacrifice working days on the farm to take care of the sick. This reduces farm productivity while also having to spend cash to seek medical care [62, 63]. In this setting, participating in MRCG activities – recognised as an effective institution for health development – is perceived to be a way to address general burdens including health, financial, and social hazards [64].

People showed a great deal of willingness to participate in the MRCG-led trial, notwithstanding low levels of concern for malaria or limited understanding of what the trial was about. This indicates that trial specificities matter less than having MRCG in their village, because of the perceived benefits stemming from good care and free medication through MRCG trials [65, 66]. Moreover, with its longstanding history of providing health services and employment to many Gambians [67], participating in MRCG activities is believed to lead to job opportunities, health care access, and the other assets it holds, such as prestige, recognition, trust, and wealth. Being a MRCG study village was considered *“very lucky”* by most respondents, as being on the MRCG map is a first step leading to the benefits associated with it.

You (MRCG) just always have your minds here. That is what we need, just do not forget about us. Always your minds be on us. – Man, village-15

Therefore, since gaining social recognition and prestige were considered beneficial for the community, it led to motivation to participation [58]. However, as Boyart reminds us, distribution of and access to benefits is highly inegalitarian and hierarchical [68]. Thus, we carefully note that collectively pursued communal benefits might not be equally distributed in the community.

### VHWs as health diplomats

Health is not only at the main hospitals, but health starts at the communities. [ … ] It’s not easy for the doctors to identify all sick persons in the communities. If there is a representative in the village who can support [sick persons] is good. – VHW, village-17

VHWs are an important link between communities and health systems. Often they are perceived as being a ‘facilitator’, ‘mediator’, or ‘broker’ to deliver health care on behalf of health systems or vertical health interventions [6, 14, 69]. In our study, we preferred to view the representation of VHWs as ‘on behalf of the communities’ than ‘on behalf of health systems/interventions’. VHWs represented their communities; they negotiated with the MRCG to obtain community benefits, adequately informed local populations, and tried to minimise health-related issues experienced in their villages. Because of these qualities [70] and capabilities, we consider viewing VHWs as *health diplomats*, rather than health-facilitators, −mediators, or -brokers for selective health interventions.

#### Political selection and positioning of VHWs

The selection of VHWs is an important factor that touches on the failure or success of the primary health care [26]. The selection criteria and process are context specific. In some cases having education is valued for a VHW’s capability [30, 71]. In other instances, female VHWs may be preferred because patients feel more at ease disclosing health issues to women [72], or because men tend to drop out more due to higher responsibilities for farm work and family income [73].

In case of The Gambia, VHWs are selected by the *Alkalo* and village members; either the *Alkalo* suggests an individual and people vote for or against the candidate, or the community members choose an individual and the *Alkalo* (symbolically) approves. Selection criteria, according to the residents, were: local to the village (born or lived long enough in the village), active, hardworking, respectful to elders, disciplined, responsible, trustworthy (e.g. keep confidentiality, prioritise people’s health), and able to represent the village. In short, the VHWs should be candidates who can prioritise communal (*sunu*) interests over their own (*sama*). In case VHW was considered not active or representing the community, there would be a re-election of VHW with the *Alkalo*’s intervention.

“Community embeddedness”, shown to be important for successful primary health care [6, 16, 29, 74], was equally important within this study.

If [medicine] is from you (MRCG nurses/fieldworkers), we will take it. But if [the VHW] gives us the medicine, we will be more comfortable with that because he is the one we know. – Compound head, village-17

However, community embeddedness alone is insufficient to bridge the community and health system [15]*.* The capability to represent a village and to negotiate with others, such as outsiders with influential assets like the government and the MRCG, is an additional requirement of VHWs. In the rural Gambia, generally men are perceived to have these capacities.

Why we select men here is because most of our activities [are] with the government. When we delegate him, he always does his best. – Alkalo, Village-4

Moreover, in the study area, VHWs were often linked to influential personages in society such as the *Alkalo* or the head of Village Development Committee (VDC). Similarly, they often had reputations of being active in community development. In this manner, VHWs were endowed with structural advantages (e.g. having a good social network, being familiar with ‘leading’ the village, being able to negotiate) to execute their role and responsibility as health diplomats. This structural advantage was likely to allow VHWs to advocate to address health disparities and related economic, social, and political issues of communities [34]. This further allowed them to nurture their VHW skill-sets and capabilities to help the community achieve a healthy living [39, 51]. In our study, it enabled VHWs to voice concerns about the disadvantaged situations communities endure (i.e. limited access to health care services), and continuously campaign for better access to care. Despite VHWs being considered at the bottom of the hierarchical health system as village volunteers who are not fully skilled to be paid professionals [13, 75], within their communities, VHWs are highly regarded as capable representatives.

#### A pathway to political status

By highlighting the political role of VHWs, we argue that within this context becoming a VHW is a pathway to becoming an influential decision-maker in a village – amongst others such as the *Alkalo*, the VDC head, and the council of elders. However, the advocacy role of VHWs is understudied in an African context, and their aspiration to gain political recognition seldom receives attention. In fact, such aspiration is demonstrated only in implicit ways. For example, most of the VHWs in our study demonstrated their motivation to take a VHW role so as *“to contribute to the society”*. This is somewhat true, but it is also misleading to assume that VHWs are ready to sacrifice themselves unwittingly and contribute voluntary time and effort for their community. Without a nuanced exploration, symbolic incentives i.e. political recognition that VHWs pursue may be missed out. We observed a case where an older VHW refused to let go of his role despite people’s concern about his age. He claimed that: *“To keep working for the MRCG is* my *role”* (*VHW, village-1)*. Our findings suggest that this was not merely due to a pure sense of dedication to his village but rather considered as an aspiration to retain (or increase) his political status and thus symbolic capital by being linked to the MRCG.

Throughout countless interactions with VHWs in the study area, we encountered many situations where VHWs advocated community health and through these acts simultaneously claimed their political position. We witnessed a VHW actively advocating to get water pumps in the village, in front of influential community members, stating: *“You (MRCG) bring the medicine but there’s no clean water, so how can we drink this medicine?” (VHW, village-7)*. At this moment this VHW performed his role of being a responsible representative of the village and proved his ability to negotiate with the MRCG, and act upon *sunu* (collective) benefits. Another VHW complained to us on behalf of his village residents about the trial, while at the same time promoting his village and assuring to be on the MRCG map: *“I am sure this village will have more turn out than other villages”* (VHW, village- 2). These cases exemplify VHWs simultaneously playing both communicator and negotiator roles to represent their community’s interests. Furthermore, this interaction with MRCG reinforced the symbolic capital of VHWs within their communities.

Now people see the MRC[G] staff coming to collect data from me. They have more trust [in me] now because they are seeing (MRCG) doctors coming to me. – VHW, village-7

Nonetheless, going up the political ladder does not happen in an instant. For instance, a relatively young (~late 20s) and newly appointed VHW usually relied on higher powers and the hierarchy above him to enhance his role.

Whenever I want to summon a meeting, I inform *Alkalo* about it. […] You know if I mention my name probably many would not come. So, if I want a meeting, I just go to the *Alkalo* and inform him about it. [Then] meetings [are] always successful. – VHW, village-4

#### Health diplomats and its impacts

To serve as functional health diplomats who advocate to address health disparities, VHWs should be able to perform social and medical roles, such as mobilising local populations to receive antenatal care, vaccination, or malaria treatment [7]. Indeed, VHWs’ advocacy should be viewed as a form of caregiving within the existing primary health care [35]. To fulfil this multifaceted role, VHWs must be equipped with adequate tools [17], which in our case were malaria diagnostic and treatment tools. Data from both the qualitative and quantitative strands indicate there is a likelihood that given the right tools and skills, alongside a positive perception of the VHWs by community residents, VHWs improved performance on their tasks, and as a result increased the participation of the community in the trial. It points to the VHWs contribution in ameliorating the economic impact on people in seeking care from farther away health centres to reduce vulnerability in the communities. All quantitative results below show response (people’s perception) changes in percentage from baseline to endline, respectively.

Table 1 shows that respondents who see VHW “always” and “most of the times” has rapid-diagnostic test (RDT) increased from 21 to 58%, while “never” decreased from 35 to 5%. Statistically, it is very likely that the responses are strongly related before and after the intervention, supporting the positive change in people’s perception towards VHWs due to the intervention. Similar figures are present for VHW having antimalarial drugs: VHW “always” and “most of the times” has antimalarials went up from 26 to 60% while “never” went down from 12 to 1%.

**Table 1:** People’s perception on VHWs with RDTs and antimalarials

Does the VHW have RDT?	Baseline	Endline	Total
Always	14.6	23.9	(35)
Most of the times	6.7	33.7	(37)
Sometimes	18.0	26.1	(40)
Never	34.8	5.4	(36)
Don’t know (DK)	25.8	10.9	(33)
N (100%)	89	92	181
Chi-square = 44.7 (df = 4); prob. < 0.0001; Cramer’s V = 0.50 (missing: 49)			
Does the VHW have antimalarials?	Baseline	Endline	Total
Always	12.2	28.4	(38)
Most of the times	13.3	31.6	(42)
Sometimes	46.7	30.5	(71)
Never	12.2	1.1	(12)
DK	15.6	8.4	(22)
N (100%)	90	95	185
Chi-square = 26.7 (df = 4); prob. < 0.0001; Cramer’s V = 0.38 (missing: 45)			

Table 2 shows that residents who said they visit the VHW when being ill rose from 40 to 64%, while “sometimes” and “never” declined from 60 to 36%. Likewise, when having a fever, people’s choices to visit the VHW among other health care services increased from 12 to 22% in contrast to a decrease in choosing health facilities from 87 to 74% (with borderline significance). It thus seems likely that the population began to perceive VHWs as functional health providers with adequate tools whom they can seek initial health care from. In case of persistent fevers, nobody (in the small sample size) responded to seeking care from VHWs for both baseline and endline, indicating that people tend to seek a higher level of care for prolonged illnesses.

**Table 2:** People’s choices of health care destination

When you are ill, do you go to the VHW?	Baseline	Endline	Total
Always	17.7	29.2	(45)
Most of the times	21.9	34.4	(54)
Sometimes	50.0	27.1	(74)
Never	10.4	9.4	(19)
DK	-	-	-
N (100%)	96	96	192
Chi-square = 11.9 (df = 3); prob. < 0.01; Cramer’s V = 0.25 (missing: 38)			
Where do you go in case of fever?	Baseline	Endline	Total
Traditional healer/Marabout	0.8	2.0	(3)
VHW	12.0	22.0	(37)
Health facility	86.4	76.0	(184)
DK	0.8	0.0	(1)
N (100%)	125	100	225
Chi-square = 5.51 (df = 3); prob. < 0.14; Cramer’s V = 0.17 (missing: 5)			
In case you don’t recover, where do you go next?	Baseline	Endline	Total
Traditional healer/Marabout	2.4	1.0	(4)
VHW	0.0	0.0	(0)
Health facility	96.8	99.0	(220)
DK	0.8	0.0	(1)
N (100%)	125	100	225
Chi-square = 1.42 (df = 3); not significant; Cramer’s V = 0.08 (missing: 4) * *the null (0.0) cells are due to small sample size*			

Table 3 shows people’s perception on VHWs’ availability declined from baseline to endline, suggesting that the trial interventions did not improve or change VHWs’ situation as busy farmers/herders. Moreover, considering the seasons when the two surveys were administered – just prior to the rainy season (baseline) and during/end of the rainy season (endline) – it is possible that community members including VHWs were occupied for the farm work which resulted in the drop seen in endline survey. People who appreciated VHW’s advice slightly improved, nevertheless not statistically significant.

**Table 3:** People’s perception on VHWs’ availability and advice

Is the VHW available when you need him?	Baseline	Endline	Total
Always	40.2	39.6	(75)
Most of the times	30.4	26.0	(53)
Sometimes	22.8	29.2	(49)
Never	3.3	1.0	(4)
DK	3.3	4.2	(7)
N (100%)	92	96	188
Chi-square = 2.26 (df = 4); not significant; Cramer’s V = 0.11 (missing: 42)			
Do you think the VHW provides good advice regarding health?	Baseline	Endline	Total
Very good	27.5	28.1	(52)
Good	60.4	67.7	(120)
Bad	1.1	0.0	(1)
No advice	6.6	1.0	(7)
DK	4.4	3.1	(7)
N (100%)	91	96	187
Chi-square = 5.5 (df = 4); not significant; Cramer’s V = 0.17 (missing: 43)			

Overall, although various “conjunctures of vulnerability” in communities lead to multiple pathways in people’s health-seeking paths [60], our results confirm that people who went to VHWs perceived receiving augmented care, which in turn we believe, contributed to improved participation in the trial.

He (VHW) is doing great work here. You know, if you are sick you can go to him or he would pack his stuff and come to your compound. He is very willing and active. That is the reason why some people who were not interested in the [trial] are now all willing to participate. – Marabout/Compound head, village-4

While well-equipped VHWs in general tend to improve access to care, they can also contribute to intra-village inequalities. VHWs, as village residents, depended on their social networks [21] that influenced whom they can or cannot reach, and consequently determined who were included or excluded from benefitting the pursuit of collective healthy living. As Green critiques, promoting community health through VHWs can be a selective empowerment [76]. The more VHWs are placed within central positions of society, the harder it can get for marginalised populations to access their services, perpetuating inequality. When individuals are excluded, their capability to participate in society is even more reduced [77], resulting in further disadvantaged situations in forming the social relationship [78]. For instance, during our study, we observed a family in one village who had migrated from Guinea and were not completely integrated into the village. According to a VHW, *“They are Fula (ethnic group) but Fula Fouta (Fula from Guinea)”* (VHW, Village-4), meaning that they are not well linked with the rest of the village. Without having a strong social network, this Guinean family was found to not be participating in village activities. Nevertheless, important to note, in general in study villages where social cohesion is highly regarded, the ethnic difference is not a barrier to VHWs providing health services to all ethnic groups. If issues arise, *Alkalo* and ethnic group representatives intervene to solve the issues.

#### Contradicting expectations

Being functional health diplomats created people’s expectations on VHWs that worked contradictorily: the first is based on the social expectation on ‘good and responsible Gambians’ who act on *sunu* and contribute to their communities; and the second is the expectation on ‘health diplomats’ who work with outside forces (e.g. MRCG) and serve their community.

As stated earlier, sharing one’s benefits is highly valued in rural Gambian communities under *sunu* and Islamic values. An act of sharing is a way to demonstrate ‘good power’ that receives prestigious recognition in return (which in some contexts reproduce inequality; see Boyart, 1993). The same logic is applied to VHWs in rural Gambia that gained symbolic capital in an exchange for contributing voluntarily to their communities. Using this reciprocal logic, the government arranged (verbally) that community members should support VHWs farm work in return for their contribution. However, this community support hardly ever happened or was sustained because people assumed VHWs to receive economic support from the government or MRCG. Indeed, in the RHOST trial VHWs naturally received a minimal financial incentive (1500Dalasi per month) for their additional work. However, all VHWs in our study claimed that even without a financial support, people would assume VHWs to receive benefits from external institutions and hence not help them on the farm.

[The government] said, “when you [become VHW] there is no payment, but the villagers would help and weed your farm for you”. But when it starts raining and you have not sown your farm, will you think of helping another person? The farm is spoiled before you realise. […] People will not [support] me because even today after you (MRCG) leave, they will think you have given me money. – VHW, village-2

Moreover, some community members perceived that VHWs were not sharing but “eating” the benefits [68] they received from the MRCG. This translated to perceptions that VHWs failed to prioritise community’s interest (*sunu ite*) over their own interest (*sama ite*), acting selfishly (*añan*), thereby undeserved to get community support.

Naturally, many VHWs expressed that they *“sacrifice”* their time, work requirements, and thus their household income for taking on this voluntary-based role. VHWs can be overwhelmed by additional workload imposed by health-related programmes [27, 42] and pressured to meet people’s expectations, especially under such contradicted expectations. The pressure seems to be also associated with the supply of medical tools, as it is said that failing to equip VHWs can disappoint people’s expectations leading to blame on the VHW [15].

Lack of medicine can cause people to stop coming to me. If I’m running out of medicine all the time, people will not come to me. […] People will perceive that I always don’t have medicine so it’s better not to go to [VHW]. – VHW, village-5

### Participatory approach

Accounting for the political status of the VHWs and integrating their socio-political dynamics in health programmes is vital for reinforcing community participation. We used an iterative and interactive community participatory approach, the Community Lab of Ideas for Health (CLIH) [43] to gain in-depth contextual insights surrounding VHWs and reasons for community participation. This approach involving constant dialogues with communities led to the nuanced understanding of the social, cultural, and political environment of communities and the positioning of VHWs in their society. We then co-designed the trial implementation strategy with communities and placed VHWs as the protagonists of trial implementation and main communicators between the MRCG and the communities. This enhanced their social and political status in the village and strengthened their medical role. It is fundamental that we understand the dynamics of the communities within which VHWs are situated and highlight the ﻿broader health system and political economy they reside in [79]. Only by addressing this larger picture, can we then start to see how the role of VHWs can truly influence and enhance community participation.

Finally, a stronger political commitment to support VHWs, not only in technical/medical support but also in their socio-political environment, is required [2, 6, 16]. It is important to coordinate and combine general health services and vertical health interventions, and vital to ensure that VHWs are not overburdened by their duties [80].

## Conclusion

In the rural Gambian context, VHWs are trusted representatives of a community who have the capacity to negotiate with external institutions and advocate a community’s healthy living. Our study showed that VHWs could contribute to reducing health-related burdens in their community, by campaigning for better access to health care services and adequately being equipped with medical tools. We noted VHWs aspiration to take a political role and furthermore acknowledged them as health diplomats – respected and valued members of their communities who are capable to bring benefits. We argue that, in the context of rural Gambia, supporting VHWs beyond medical roles but also in social and political roles contributes to the improved performance of VHWs and community participation in activities that communities perceive beneficial.

## Data Availability

The datasets generated and/or analysed during the current study are not publicly available because participants did not consent to have their full transcripts made publicly available. However, the NVivo database with excerpts of the transcripts relevant to the study is available from the corresponding author on reasonable request.

## Abbreviations

VHWs: Village Health Workers
RHOST: Reactive Household-based Self-administered Treatment against residual malaria transmission
MRCG: Medical Research Council unit in The Gambia
CLIH: Community Lab of Ideas for Health
VDC: Village Development Committee
RDTs: Rapid Diagnostic Tests
